# Effects of prenatal alcohol exposition on cognitive outcomes in childhood and youth: a longitudinal analysis based on meconium ethyl glucuronide

**DOI:** 10.1007/s00406-023-01657-z

**Published:** 2023-08-02

**Authors:** Jakob Roetner, Jessica Van Doren, Janina Maschke, Louisa Kulke, Constanza Pontones, Peter A. Fasching, Matthias W. Beckmann, Bernd Lenz, Oliver Kratz, Gunther H. Moll, Johannes Kornhuber, Anna Eichler, und das IMAC-Mind-Consortium

**Affiliations:** 1 Department of Child and Adolescent Mental Health, University Hospital Erlangen, Friedrich-Alexander University Erlangen-Nürnberg (FAU), Schwabachanlage 6, 91054 Erlangen, Germany; 2 Department of Psychology I – Developmental Psychology, Otto-Friedrich-University Bamberg, Bamberg, Germany; 3 Department of Gynecology and Obstetrics, University Hospital Erlangen, Friedrich-Alexander University Erlangen-Nürnberg (FAU), Erlangen, Germany; 4 Department of Psychiatry and Psychotherapy, University Hospital Erlangen, Friedrich-Alexander University Erlangen-Nürnberg (FAU), Erlangen, Germany; 5 Department of Neurocognitive Developmental Psychology, Friedrich-Alexander University Erlangen-Nürnberg (FAU), Erlangen, Germany; 6 Department of Addictive Behavior and Addiction Medicine, Central Institute of Mental Health (CIMH), Medical Faculty Mannheim, Heidelberg University, Mannheim, Germany

**Keywords:** Prenatal alcohol exposure, EtG, FRANCES, EEG, Event-related potentials

## Abstract

**Background:**

Prenatal alcohol exposure (PAE) has been linked to severe, adverse child outcomes. However, little is known regarding subclinical outcomes of low/moderate PAE and its longitudinal consequences, especially regarding neurophysiological and neurocognitive development. A newborn biomarker of PAE, meconium ethyl glucuronide (EtG), has been shown to predict cognitive impairments in primary-school-aged children. The current study investigated the ongoing effects of subclinical PAE in adolescence.

**Methods:**

A sample of *n* = 96 mother–child dyads of the FRAMES/FRANCES cohort were classified into PAE/no PAE using EtG with a 10 ng/g cutoff. Mothers were recruited during pregnancy and children were assessed during primary-school age (*M* = 7.57, *SD* = 0.65, *range*: 6.00–9.92 years) and adolescence (*M* = 13.26, *SD* = 0.31, *range:* 12.79–14.20 years) on three levels: clinical (ADHD rating), neuropsychological (IQ score and performance in a go/nogo task), and neurophysiological (analysis of P3 event-related potentials (ERP) during said go/nogo task). Developmental outcomes and courses following PAE were assessed using rmANCOVAs, controlling for relevant confounders (socioeconomic status (SES), birth weight, and maternal psychopathology).

**Results:**

Neurophysiological impairments emerged for exposed children in the form of diminished attentional resource recruiting in childhood and adolescence (reduced go-P3 amplitudes) with no differences in performance. Neuropsychological testing showed a reduced IQ score for both time points with dose-dependent effects in childhood. Clinical ADHD symptoms were not significantly affected.

**Conclusion:**

Subclinical PAE, as determined by meconium EtG, has negative developmental consequences on cognitive function that persist from childhood to adolescence. These findings suggest that there is no safe limit for alcohol consumption during pregnancy and that more thorough screening of alcohol consumption during pregnancy is necessary for early identification and treatment of at-risk children.

**Supplementary Information:**

The online version contains supplementary material available at 10.1007/s00406-023-01657-z.

## Introduction

Prenatal alcohol exposure (PAE) is a known risk factor for adverse fetal and child development [1], potentially leading to a spectrum of maladaptive outcomes, clustered under the term fetal alcohol spectrum disorders [FASD; 2]. Its most severe outcome is fetal alcohol syndrome (FAS), a clinical disorder often diagnosed through physical examination, e.g., of facial features [2, 3]. Epidemiological assessment of FAS and FASD is a complex topic and prevalence rates vary depending on region and assessment method, with, e.g., 6 to 9 per 1000 children (FAS) and 24–48 per 1000 children in a representative community in Midwestern US being affected [4]. However, there is growing evidence for subclinical impairments following PAE in children and adolescents which do not necessarily meet the diagnostic criteria for FASD (see e.g., [4]). This includes: cognitive deficits [6], neurophysiological or neurological changes [7–9]. The nature of these impairments is still being investigated, linking PAE with attention deficit hyperactivity disorder (ADHD) like symptoms such as attentional deficits [10], distinct patterns in hyperactivity/impulsivity [11], and reduced inhibitory control[12]. While deficits seem similar, neuronal mechanisms have been found to differ between PAE attentional impairments and non-PAE ADHD symptoms [8, 13, 14]. Additionally, the trajectories and longitudinal mechanisms of developmental PAE outcomes from (early) childhood to adolescence are poorly understood [5, 15, 16].

Despite FASD being one of the most common abnormalities at birth, with the precise diagnosis essential for early and effective care [17], it is often misdiagnosed or undiagnosed [18]. A potential solution for this is to routinely assess for PAE risk. Three possible methods for assessing the prevalence of PAE in a population have been postulated [19]: (1) assessing developmental impairments in children and adolescents which may be linked to PAE, (2) estimating PAE through self-report instruments assessing maternal alcohol consumption, and (3) using biomarkers.

Developmental impairments can be assessed at a clinical, neurophysiological, and neurocognitive levels. Clinical assessments can be made using maternal symptom reports as well as clinician observations [20]. Neurophysiological assessment of ADHD-like symptoms can be achieved using neural markers in EEG data, specifically event-related potentials (ERPs) [21]. IQ tests and behavioral tasks can be used to assess neurocognitive impairments [22]. While these assessments help to identify missed cases of PAE, the late diagnosis results in a missed opportunity for early intervention [22].

Current research using self-report measures estimates the prevalence of PAE to be between 10% [23] and 20% [6, 24]. However, these numbers may be significantly (up to fourfold) underreported [19, 25]. Therefore, maternal self-report should be used cautiously as an accurate indicator of PAE.

In an effort to address these problems, ethyl glucuronide (EtG), a biomarker for PAE, has been used to assess both low and medium levels of PAE in previous studies [3, 19, 26–28]. EtG is an ethanol metabolite which can be analyzed through the meconium (first stool of the child), reflecting primarily PAE during the third trimester of pregnancy. The impact of its effects on development, cognition, and neurophysiology in primary-school-aged children has been investigated by our research group using this method [8].

Our findings indicate that there are PAE-specific attention-related neurophysiological deficits which differ from those of children with ADHD symptoms but without PAE. Additionally, we found lower IQ scores in children exposed to alcohol. However, such adverse effects of PAE are influenced by risk and protective factors throughout development [29] and need to be assessed longitudinally. Therefore, the aim of this study is to evaluate the effects of PAE (clinical, neuropsychological, and neurophysiological) from primary-school age to adolescence. For significant EtG results, the predictive value of the maternal pregnancy self-reports will be comparatively tested. Additionally, within the EtG-positive group, dose–response effects will be examined. We hypothesized that (1) PAE shows a longitudinal effect (primary-school age to adolescence) with EtG-positive children showing impairments/differences in clinical (i.e., ADHD symptoms), neurophysiological (i.e., ERP differences), and neuropsychological (i.e., IQ, go/nogo performance) domains, (2) there are dose–response effects within the EtG-positive group with higher dosages leading to more severe outcomes, and (3) the maternal self-report does not necessarily match with the results of biomarker analysis, since we deemed them as not as reliable. This study will be an important addition to enhance the understanding of PAE and its mechanisms further, especially since it is one of the few longitudinal studies looking at a multitude of different outcomes in young children and adolescents.

## Methods

### Study design and sample definition

The present work is a cooperation between the Departments of Obstetrics and Gynecology, Psychiatry and Psychotherapy, and Child and Adolescent Mental Health at the University Hospital Erlangen, Germany. The initial assessment (Franconian Maternal Health Evaluation Study (FRAMES)) was performed at the Department of Obstetrics and Gynecology from 2005 to 2007 [30, 31]. Women (*n* = 1100) were recruited during their third trimester of pregnancy. Between 2012 and 2015, a subsample of these women (*n* = 618) was contacted for participation in a follow-up study and *n* = 245 FRAMES mother–child dyads (39.6%; child age: *M* = 7.74 years, *SD* = 0.74) took part in the FRANCES I study (Franconian Cognition and Emotion Studies) at the Department of Child and Adolescent Mental Health [19, 32]. The mothers and children were contacted again from 2019 to 2021 to take part in the second follow-up of the study (FRANCES II). Of 245 contacted families, 186 (75.9%) agreed to participate again (child age: *M* = 13.3 years, *SD* = 0.34, *range*: 12.8–14.5). When comparing participating families with non-participating families, no significant differences in marital status (*χ*^2^(1) = 0.35, *p* = 0.552), family income (*χ*^2^(4) = 3.94, *p* = 0.414) or maternal total psychopathology (*t*(234) = − 0.93, *p* = 0.353; definition: see below) at time of FRANCES I were found. Additionally, no association between dropout and EtG status could be found (*χ*^2^(1) = 1.19, *p* = 0.278). However, mothers with a higher education were more often willing to re-participate (*χ*2(1) = 7.60, *p* = 0.006). In FRANCES I and FRANCES II, multiple parameters were evaluated following a multi-level design (clinical, neurophysiological, neuropsychological, neurobiological) to assess child outcomes in terms of cognitive, emotional, and social development. The study was approved by the ethics commission of the Friedrich Alexander-University Erlangen-Nürnberg (FAU) and conducted in accordance with the Declaration of Helsinki. All participants provided informed consent/assent.

For defining the sample of the given study, all participants with successfully recorded neurophysiological and performance data during a go/nogo task from FRANCES I (*n* = 215) were assessed for additional exclusion criteria: (1) no participation in FRANCES II (*n* = 48, 22.3%), (2) no EEG recorded in FRANCES II (*n* = 43, 20.0%), (3) no valid EtG level from FRAMES (*n* = 17, 7.91%), (4) methylphenidate use during FRANCES I and/or FRANCES II testing (*n* = 1, 0.47%), (5) missing FRANCES II neurophysiological or performance data (*n* = 10, 4.65%). This led to a final sample of *n* = 96 participants for further analysis.

### Measurement of prenatal alcohol exposition

Meconium was collected and analyzed within 2–24 h after birth, with EtG analysis applied as described by Bakdash et al. [27]. Children were classified as exposed to prenatal alcohol (EtG +), if meconium EtG levels were ≥ 10 ng/g (detection limit). Children negative for PAE will be referred to as EtG-. Maternal self-report of PAE was assessed via an interview conducted by well-trained medical assistants during the third trimester of pregnancy. Participant answers were categorized as PAE negative (‘I don’t drink in general’ and ‘I didn’t drink during pregnancy’) or PAE positive (‘I rarely drank during pregnancy’ and ‘I drank one glass/day during pregnancy’). Please note: while the two items classified as PAE positive imply a large difference in alcohol consumption (rarely drinking vs. daily drinking), they were grouped together anyway, since nearly no participants reported daily drinking (*n* = 1).

### Clinical ADHD measures

ADHD-related behavior of the children was measured through maternal rating. For FRANCES I, the *German ADHD rating scale—second edition* [33] was used; FRANCES II used the third edition [34]. Both instruments feature 20 items (4-point Likert scale) and provide a total score (ADHD_total_) as well as 2 subscale scores ‘inattention’ (ADHD_IA_) and ‘hyperactivity/impulsivity’ (ADHD_hyp/imp_), with a conceptualized range from 0.00 (‘not at all’) to 3.00 (‘notably’).

### Neurophysiological measures

**Fig. 1:**
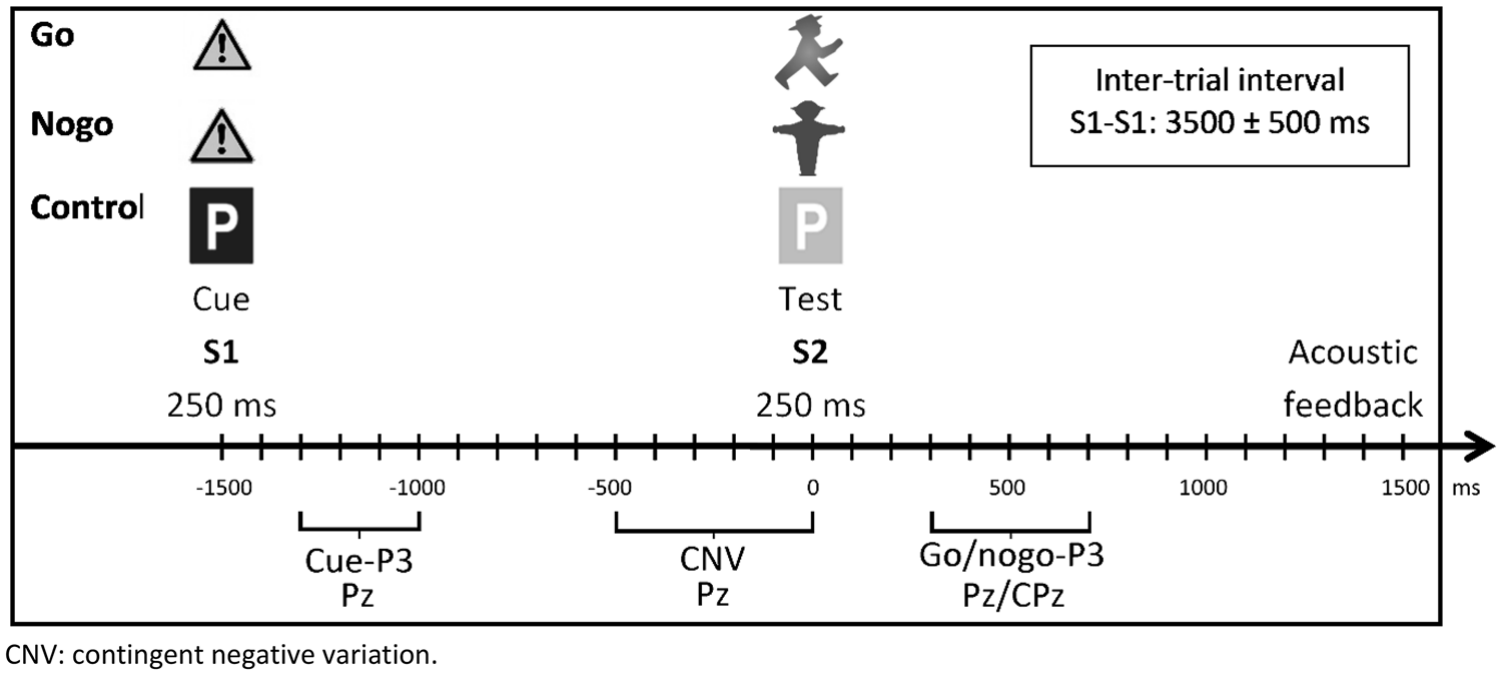
Schematic illustration of the cued go/nogo task (S1–S2 paradigm). CNV: contingent negative variation

Neurophysiological impairments were measured using a go/nogo task (see Fig. 1) implemented with presentation (Neurobehavioral Systems, Albany, CA) as described by Eichler et al. [8]. The task consisted of 4 blocks with 36 trials per block. Each trial started with the presentation of a cue stimulus that was followed by a test stimulus. Go, nogo, and control trials were shown with equal probability. In the second and third task block, a monetary reward was given for fast responses in go-trials. In the present work, blocks were averaged regardless of reward.

Recording and processing of EEG were performed identically to that of Eichler et al. [8] and are described here briefly (details see Supplement S1). Segments were excluded from further analysis if: (1) they included amplitudes outside of a ± 150 μV range, (2) the participant made a performance error (e.g., reaction in a nogo-trial), and (3) in case of a go-trial, the participant did not respond between 200 and 1500 ms after the S2 stimulus. Participants were excluded if less than 50% of segments remained for any of the four conditions (go/nogo, with/without incentives) after these quality control steps (*n* = 14). Finally, the following ERP components were defined as described by Eichler et al. [8]: CNV (contingent negative variation; mean amplitude from -500 to 0 ms; Pz) and cue-P3 (maximum amplitude within − 1300 to − 1000 ms; Pz), go-P3 (maximum amplitude within 300–700 ms; Pz), and nogo-P3 (maximum amplitude within 300–700 ms; CPz).

### Neuropsychological measures

To assess neuropsychological development, the intelligence quotient (IQ) was assessed in FRANCES I using the standardized Intelligence and Development Scales [IDS; 35] and in FRANCES II using the Wechsler Intelligence Scale for Children—Fifth Edition [WISC-V; 36]. Total IQ scores have been calculated based on age- and gender-specific norms (*M* = 100, *SD* = 15). The performance during the described go/nogo task has been operationalized through the following measures: mean reaction time (RT_M_); reaction time variability (RT_STD_); number of impulsivity errors (ERR_imp_).

### Confounders and additional parameters

We controlled our analysis for socioeconomic status (SES), birth weight, and maternal psychopathology. The SES was based on FRANCES I reports of both maternal and paternal education level (operationalized via years in school; 4 level: < 9, 9, 10 or 13 school years) and net family income per month (6 level: < 1000 € to > 5000 €) and was calculated as a sum score (theoretical range: 3–14 points). The birth weight was registered in grams immediately after delivery. Maternal psychopathology was assessed at FRANCES I and FRANCES II with the *Brief Symptom Inventory* [BSI; 37]; the Global Severity Index (*T* score: *M* = 50, *SD* = 10) from both time points was averaged.

### Statistical analysis

The analyses used *IBM SPSS Statistics*, version 24.0 (IBM Corporation, Armonk NY; USA). The level of significance of all analyses was defined as *p* < 0.05 (two tailed). For the analysis of clinical (ADHD), neurophysiological (ERPs) and neuropsychological (IQ, performance) data, participant EtG status (EtG + vs EtG−) was used as between-subject factor in repeated measure ANCOVAs (rmANCOVAS), with the time point of the measures (FRANCES I vs FRANCES II) used as a within subject factor. Additionally, the interaction *time point x EtG* has been added. The above given confounders have been controlled as covariates. Partial eta squared (η_p_^2^) values are reported as the effect size measure. If a significant EtG−/ + effect emerged, the same rmANCOVA was run using the factor ‘PAE positive vs. PAE negative’ according to prenatal maternal self-reports.

To evaluate potential dose–response effects, partial correlations (confounder-controlled correlations between EtG level and measured clinical, neurophysiological, and neuropsychological outcomes) were run within the EtG-positive group. For this analysis, continuous EtG values ≥ 10 ng/g were log-transformed, since they were not normally distributed (Shapiro–Wilk test: *W*(25) = 0.47, *p* < 0.001).

## Results

### Sample characteristics

From the *n* = 96 participants, 26.04% (*n* = 25) were considered as EtG + (meconium level ≥ 10 ng/g) with absolute EtG values of the EtG + group ranging from 17 ng/g to 2400 ng/g (*M* = 260.52, *SD* = 471.52). The children were *M* = 7.57 (*SD* = 0.65, *range*: 6.00–9.92) years old at FRANCES I and *M* = 13.26 (*SD* = 0.31, *range:* 12.79–14.20) years old at FRANCES II. Only at the elementary school time point, the EtG + children were slightly older (*p* = 0.028). Mothers’ alcohol consumption self-report was associated with EtG status, *X*^2^(1) = 4.465, p = 0.035, ϕ = 0.22. There were no EtG + to EtG− group differences in birth weight, SES, maternal psychopathology or adolescent age. An overview of all sample characteristics can be found in Table 1.

**Table 1:** Sample characteristics

	EtG + N = 25	EtG− N = 71	Statistics	
	M (SD)/N (%)	M (SD)/N (%)	*t* (df)/*χ*^*2*^* (*df*)*	*d*/ϕ
Child				
EtG value [ng/g]	260.52 (471.52)	0.00 (0.00)	–	–
Gestational age [wks]	39.48 (1.45)	39.46 (1.21)	− 0.05 (94)	− 0.01
Age [years]				
FRANCES I	7.84 (0.72)	7.47 (0.60)	− 2.29 (36.47)*	− 0.58
FRANCES II	13.35 (0.37)	13.23 (0.27)	− 1.47 (33.66)	− 0.40
Birth weight [g]	3637.80 (542.28)	3445.07 (430.60)	− 1.79 (94)	− 0.42
Sex				
Male	12 (48.0%)	41 (57.7%)	0.71 (1)	0.09
Female	13 (52.0%)	30 (42.3%)		
Mother				
Age at birth [years]	32.92 (3.83)	32.94 (4.56)	0.02 (94)	0.01
Alcohol self-report (N = 90)				
Yes	10 (41.7)	13 (19.7)	4.47 (1) *	0.22
No	14 (58.3)	53 (80.3)		
BSI score				
FRANCES I	51.36 (15.04)	46.57 (13.35)	− 1.49 (93)	− 0.34
FRANCES II	42.78 (10.59)	45.39 (11.87)	0.847 (82)	0.23
SES (sum score)	11.88 (1.79)	11.66 (2.20)	− 0.45 (94)	0.10

EtG: ethyl glucuronide; BSI: Brief Symptom Inventory [37]; SES: Social Economic Status. Missings: BSI FRANCES I *n* = 1; BSI FRANCES II *n* = 12. *p < .05

### Clinical ADHD results

There was no effect of time point (FRANCES I vs. FRANCES II) for any of the ADHD measures (*p* = 0.062−0.188). EtG status did not result in any significant ADHD differences (*p* = 0.128−0.941) and no EtG x time point interaction effects were present (*p* = 0.344−0.997).

### Neurophysiological results

**Fig. 2:**
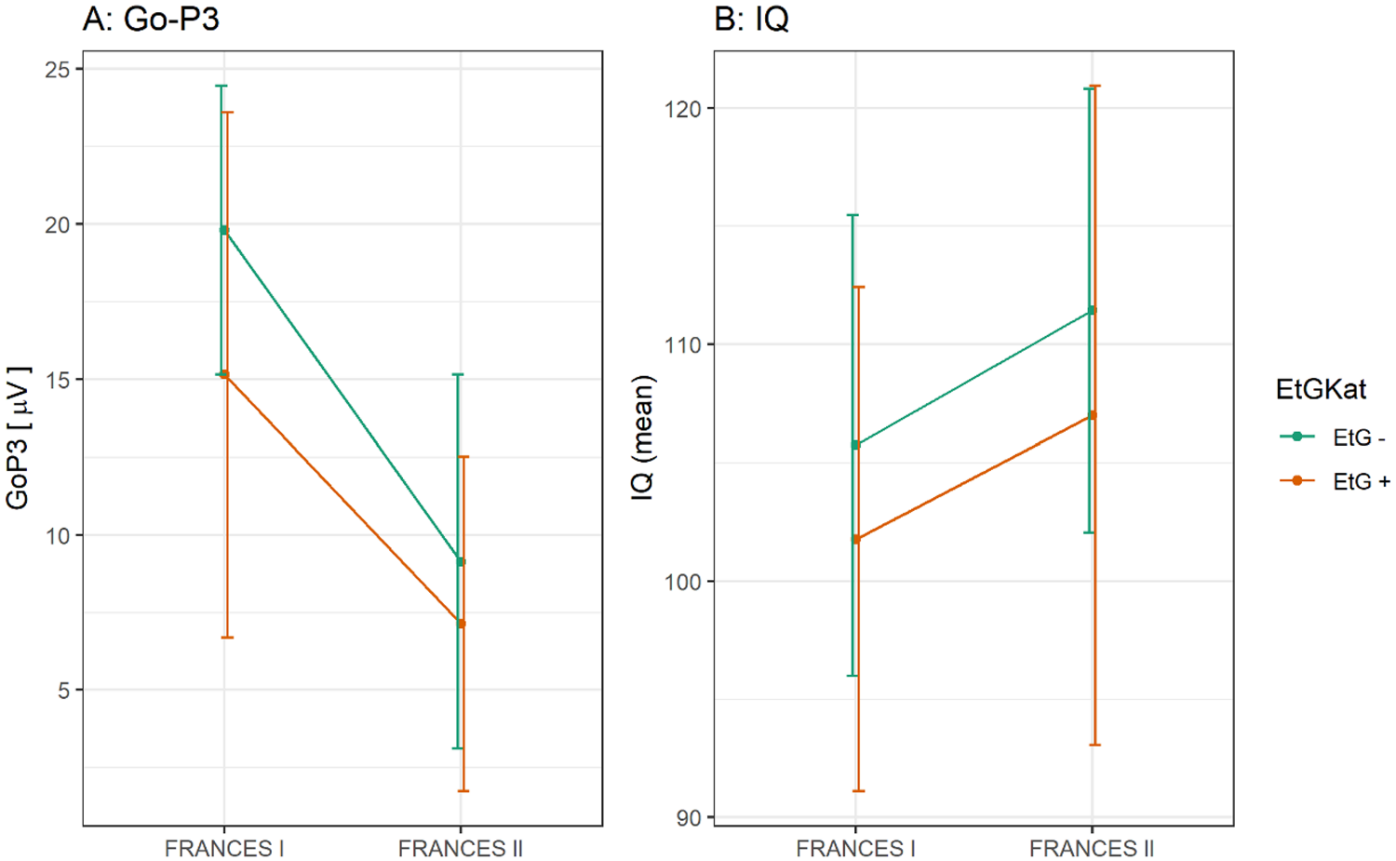
Main effects of between-subject ANCOVAs significant for Go-P3 (µV) and IQ (mean) based on EtG status. EtG + : ethyl glucuronide positive (> 10 ng/g), EtG−: ethyl glucuronide negative (< 10 ng/g); FRANCES: Franconian Cognition and Emotion Studies; IQ: intelligence quotient, total score, assessed using the standardized Intelligence and Development Scales (IDS; [35]) in FRANCES I and the Wechsler Intelligence Scale for Children—Fifth Edition (WISC-V; [36]) in FRANCES II

There was no effect of time point for any of the EEG measures (*p* = 0.184−0.926). When separated into EtG + or EtG− groups, an effect emerged exclusively for the Go-P3 component (*F*(1,77) = 5.72, *p* = 0.019, η^2^_p_ = 0.069) in which the EtG + (*n* = 20) participants had a lower Go-P3 (mean values of both time points as reported in the ANCOVA) (*M* = 11.08 µV, *SE* = 1.24) than the EtG− (*n* = 62) participants (*M* = 14.50 µV, *SE* = 0.70). Classification using the self-report of the mothers showed no significant differences in Go-P3 amplitudes (*F*(1,73) < 0.001, *p* = 0.999, η_p_^2^ < 0.001). Go-P3 data can be seen in Fig. 2.

### Neuropsychological results

The total IQ score did not differ between time points (*p* = 0.905) and no interaction effect was found (*p* = 0.803). However, a between-subjects effect (mean values of both time points as reported in the ANCOVA) was found for IQ score (*F*(1,91) = 5.70, *p* = 0.019, η_p_^2^ = 0.059): EtG + (*n* = 25) participants had a lower IQ (*M* = 103.74, *SE* = 1.82) than EtG− (*n* = 71) participants (*M* = 108.81, *SE* = 1.07). A subsequently performed analysis using the maternal self-report as between-subject factor showed no IQ differences between children from mothers who reported PAE and children from mothers who did not report alcohol consumption (*p* = 0.142). IQ data can be seen in Fig. 2.

Evaluating the performance of children and adolescents during the go/nogo task showed a significant time-point effect for RT_M_ (*F*(1,91) = 10.69, *p* = 0.002, η_p_^2^ = 0.105) and RT_SD_ (*F*(1,91) = 4.78, *p* = 0.031, η_p_^2^ = 0.050), but not for ERR_imp_ (*p* = 0.810). Children (mean values of both EtG groups as reported in the ANCOVA) (RT_M_: *M* = 468.45 ms, *SE* = 10.42; RT_SD_: *M* = 114.16 ms, *SE* = 36.78) were significantly slower and more variable in their response than adolescents (RT_M_: *M* = 306.14 ms, *SE* = 3.88; RT_SD_: *M* = 68.54 ms, *SE* = 19.87). This developmental effect is expected due to brain development and was assessed here to approximate validity of the other findings. Besides, no other significant main or interactioneffects have been found. EtG status was not associated with the performance data—neither overall (*p* = 0.124−0.467) nor at any specific time point (*p* = 0.089−0.215). All rmANCOVA results can be found in Table 2.

**Table 2:** Summary of rmANCOVA results from clinical, neuropsychological, and neurophysiological data analysis

Measure	Status	N	Descriptives					Statistics							
			FRANCES I		FRANCES II			Time point			EtG			EtG x time point	
			*M*	*SD*	*M*	*SD*	*F*	*p*	η_p_^2^	*F*	*p*	η_p_^2^	*F*	*p*	η_p_^2^
ADHD_total_	EtG +	25	0.57	0.43	0.48	0.39	3.57	0.062	0.038	0.82	0.367	0.009	0.46	0.498	0.005
	EtG−	71	0.52	0.35	0.38	0.31									
ADHD_IA_	EtG +	25	0.73	0.67	0.74	0.55	1.96	0.165	0.021	2.36	0.128	0.005	0.91	0.344	0.010
	EtG−	71	0.62	0.36	0.53	0.44									
ADHD_hyp/imp_	EtG +	25	0.48	0.39	0.27	0.29	1.76	0.188	0.019	0.005	0.941	0.000	00.00	0.997	0.000
	EtG−	71	0.47	0.43	0.25	0.32									
IQ	EtG +	25	101.76	10.65	107.00	13.94	0.01	0.905	0.000	5.70	0.019*	0.059	0.06	0.803	0.001
	EtG−	71	105.73	9.75	111.44	9.38									
Cue-P3	EtG +	62	8.63	3.20	5.41	2.73	0.347	0.557	0.004	0.970	0.328	0.012	0.737	0.393	0.009
	EtG−	20	9.49	4.14	5.52	2.97									
CNV	EtG +	62	− 5.09	2.00	− 4.38	1.54	0.009	0.926	0.000	0.491	0.486	0.006	0.010	0.922	0.000
	EtG−	20	− 5.17	2.16	− 4.58	1.40									
Go-P3	EtG +	62	15.15	4.65	7.13	5.39	1.80	0.184	0.023	5.72	0.019*	0.069	1.36	0.248	0.017
	EtG−	20	19.81	8.46	9.13	6.02									
NoGo-P3	EtG +	62	12.99	6.02	8.63	4.39	0.248	0.620	0.003	0.744	0.391	0.010	0.153	0.697	0.002
	EtG−	20	13.84	5.73	8.95	6.10									
RT_M_	EtG +	25	447.07	96.23	306.18	27.53	10.69	0.002**	0.105	2.19	0.142	0.024	2.07	0.154	0.022
	EtG−	71	486.63	88.30	306.59	34.57									
RT_STD_	EtG +	25	100.00	40.31	68.89	16.09	4.78	0.031*	0.050	2.41	0.124	0.026	2.96	0.089	0.032
	EtG−	71	119.14	34.41	68.42	21.15									
ERR_imp_	EtG +	25	0.75	0.86	0.68	0.73	0.06	0.810	0.001	0.53	0.467	0.006	1.56	0.215	0.017
	EtG−	71	0.69	0.83	1.05	1.53									

ADHD symptom range 0–3. IQ total score norm M = 100, SD = 15. EEG measures reported in µV. CNV: contingent negative variation, ERR_imp_: impulsivity errors, EtG: ethyl glucuronide, FRANCES: Franconian Cognition and Emotion Studies, RT_M_: mean reaction time (ms), RT_STD_: reaction time variability (ms), Controlled for socioeconomic status, birth weight, maternal current psychopathology*:* 1,91 for all analysis. *p < .05, **p < *.01*

### Dose–response analysis

The dose of EtG showed no significant correlation with ADHD scores, ERPs or performance data. However, a significant negative correlation between EtG concentrations and the IQ score at primary-school age could be found (*r* = − 0.52, *p* = 0.013): Higher EtG levels at birth were associated with lower IQ scores in primary-school age. However, this effect was not present for adolescence (*r* = − 0.21, *p* = 0.330). For a summary of dose–response statistics, please see Table 3.

**Table 3:** Correlational results for a dose–response effect for the EtG + group (*n* = 18–25) on measured outcomes

		**Outcome**										
		Clinical			Neurophysiological				Neuropsychological			
		ADHD_tot_	ADHD_IA_	ADHD_hyp/imp_	Cue-P3	CNV	Go-P3	Nogo-P3	IQ	RT_M_	RT_STD_	ERR_Imp_
FRANCES I	*r*	0.28	0.28	0.19	0.11	− 0.01	− 0.23	0.19	− **0.52**	− 0.22	0.06	0.33
	*p*	0.195	0.202	0.385	0.649	0.947	0.361	0.447	0.013^*^	0.317	0.789	0.127
FRANCES II	*r*	0.07	0.10	0.01	− 0.14	0.25	− 0.14	0.06	− 0.21	0.11	0.00	0.07
	*p*	0.741	0.636	0.947	0.582	0.319	0.585	0.796	0.330	0.614	0.997	0.728

EtG: ethyl glucuronide, FRANCES: Franconian Cognition and Emotion Studies, CNV: contingent negative variation, RT_M_: mean reaction time, RT_STD_: reaction time variability, ERR_imp_: impulsivity errors. EtG log10-transformed. Partial correlations controlled for socioeconomic status, birth weight, maternal current psychopathology. Significant results in **bold**. *p < .05

## Discussion

This study used a subsample of the FRAMES/FRANCES cohort trying to analyze developmental differences between PAE-positive and PAE-negative participants, with one of the aims being to replicate the work of Eichler et al. (2018). This succeeded with showing persistent developmental differences (Go-P3 and IQ) from childhood to adolescence in PAE-positive compared to PAE-negative participants. The perinatal EtG meconium biomarker hints at potential cognitive impairments over 6 to 14 years, while maternal self-reports had no predictive power. The observed subclinical cognitive impairments are of relevance to the affected individuals, restricting the developmental resources of the child—even if affected children were not ‘visibly’ pathological.

No effects could be found for ADHD symptoms after correcting for relevant confounders, neither in the whole sample nor analyzing dose-dependent differences in ADHD scores. The lack of dose-dependent effects found here may be due to the small number of highly exposed participants (*n* = 11) or potentially reflect that the cohort had ADHD-like subtype differences that are less visible in adolescence [38, 39].

Neurophysiologically, a different picture emerges: EtG + participants showed reduced Go-P3 amplitudes in contrast to EtG− participants. The lack of effects regarding time point suggest that this PAE effect can be considered age independent, and therefore stable from childhood through adolescence. This finding implicates that developmental differences seen during childhood persist into adolescence, despite the lack of reported clinical symptoms by the parents. This would be of particular importance since a reduced P3 indicates impaired attentional allocation and executive response control. This is in line with previous research that found lower P3 values for typically developing children with higher ADHD-like symptoms [40] and that PAE is related to decreased functional connectivity of the attentional networks [41].

Neuropsychological developmental deficits were found as an age-independent reduction of the total IQ score; however, a dose-dependent correlation effect with a higher EtG dose related to a lower IQ score was only found in childhood. This indicates a larger impact of PAE on neuropsychological development during childhood, but the persistent lower IQ score found for adolescents suggests that the deficits found early in life do have lasting implications. Previous studies have also found decreased cognitive function based on prenatal alcohol exposure [42, 43]. The performance of the go/nogo task of the children was subject to a developmental effect, with children being slower and showing more variability in their reaction time than adolescents. This is an expected outcome, which is congruent with previous research [44, 45]. All in all, our first two hypotheses could only be confirmed partially, since not all three proposed domains (clinical, neurophysiological, and neuropsychological) seem to be affected.

There was a relationship between EtG status and mother´s self-report which previously did not reach significance [8]. In the present study, the correlation was statistically significant (theoretically leading to the dismissal of hypothesis 3) but—oriented at the effect size measure—practically of small relevance. Eichler et al. [9] describe the association of maternal self-report in correspondence to biomarker results. Accordingly, mothers’ self-report did not serve as a predictor for cognitive impairment which is likely due to miss-reporting by the mothers [19, 25, 46].

Several limitations of the given study should be discussed: first, the assessment of ADHD-like behavior has only been drawn from parental reports and a single neurophysiological test (go/nogo task). Second, future studies should implement a broader array of tests, to assess other cognitive functions typically impaired in children with FASD (e.g., executive function tests, emotional regulation). Third, the impact on everyday life cannot be assessed with this study alone, since it mainly uses parental reports and the go/nogo task to operationalize neurophysiology and behavior. Gathering other variables, e.g., school outcomes, clinical evaluation of the children by trained professionals, etc., could be a valuable tool to analyze effects on everyday life and assess clinical outcomes. Finally, all implications regarding alcohol consumption need to keep in mind that EtG is a biomarker for PAE in the third trimester of pregnancy. Therefore, information about alcohol consumption at the earlier stages of pregnancy are missing from this analysis.

## Conclusion

In view of our results, our previously proposed three-step model [8] needs to be adjusted. Originally it was proposed that an ‘invisible’ neurophysiological reduction in attentional resources combines with an impaired cognitive test performance (‘visible to the clinician’) and culminates in ADHD-like behavior, which is then ‘visible to the mother’. The current findings suggest that the potential alterations in neurophysiology and neuropsychological impairments (IQ) might be ‘invisible’ and persist through childhood until adolescence, while behavioral measures (performance and ADHD-like behavior) are prone to developmental improvement. However, it might be difficult to draw conclusions for everyday life performance, since this study does mainly reflect measures of attention, which is not the only factor playing into behavioral deficits or deficits in executive function. Based on these findings, there is no safe limit for alcohol consumption during pregnancy and PAE should be routinely assessed to identify and provide early treatment for at-risk children. In clinical practice, maternal reports—explicitly relevant for alcohol consumption in early pregnancy—and biological markers of intrauterine ethanol exposure should be combined.

### Supplementary Information

Below is the link to the electronic supplementary material.

Supplementary file 1: (DOCX 19 KB)

## Data Availability

If you want to access data and material, please contact anna.eichler@uk-erlangen.de.
