# Supplementary material for: Effects of prenatal alcohol exposition on cognitive outcomes in childhood and youth: a longitudinal analysis based on meconium ethyl glucuronide
Source: Eur Arch Psychiatry Clin Neurosci. 2023 Aug 2;274(2):343–52. doi: 10.1007/s00406-023-01657-z (PMC10914883; doi:10.1007/s00406-023-01657-z)
Supplement: Supplementary file 1 — (DOCX 19 KB) [file 406_2023_1657_MOESM1_ESM.docx]

**Effects of prenatal alcohol exposition on cognitive outcomes in childhood and youth: A longitudinal analysis based on meconium ethyl glucuronide.**

*European Archives of Psychiatry and Clinical Neuroscience*

Jakob Roetner^1,2*^, Jessica Van Doren^1*^, Janina Maschke^1^, Louisa Kulke^5^, Constanza Pontones^3^, Peter A. Fasching^2^, Matthias W. Beckmann^2^, Bernd Lenz^4,6^, Oliver Kratz^1^, Gunther H. Moll^1^, Johannes Kornhuber^4+^, Anna Eichler^1+^ und das IMAC-Mind-Consortium

^*^ shared first authorship, ^+^ shared senior authorship

^1^ Department of Child and Adolescent Mental Health, University Hospital Erlangen, Friedrich-Alexander University Erlangen-Nürnberg (FAU), Erlangen, Germany,

^2^ Department of Psychology I – Developmental Psychology, Otto-Friedrich-University Bamberg, Bamberg, Germany,

^3^ Department of Gynecology and Obstetrics, University Hospital Erlangen, Friedrich-Alexander University Erlangen-Nürnberg (FAU), Erlangen, Germany,

^4^ Department of Psychiatry and Psychotherapy, University Hospital Erlangen, Friedrich-Alexander University Erlangen-Nürnberg (FAU), Erlangen, Germany,

^5^ Department of Neurocognitive Developmental Psychology, Friedrich-Alexander University Erlangen-Nürnberg (FAU), Erlangen, Germany

^6^ Department of Addictive Behavior and Addiction Medicine, Central Institute of Mental Health (CIMH), Medical Faculty Mannheim, Heidelberg University, Mannheim, Germany

# **Correspondence to**

PD Dr. phil. Anna Eichler, Department of Child and Adolescent Mental Health, University Hospital Erlangen, Schwabachanlage 6, D-91054 Erlangen, Phone: +49 9131 85 39122, Fax: 09131 85-39126, E-Mail: anna.eichler@uk-erlangen.de

*Supplement S1 – Overview of go/nogo-task*

*Task Implementation*

- Implemented with Presentation (Neurobehavioural Systems, Albany, CA)
- Four blocks with 36 trials per block, each trial consisting of conditioning (cue) stimulus, followed by test stimulus
- Go, nogo and control trials occurring with equal probability
- ABBA-design:
  - In the second and third task block (B), a monetary reward (10 cents per trial) was given for fast responses in go-trials to increase motivation.
  - Fast responses were defined dynamically using a tracking algorithm (75^th^ percentile of reaction times in go trials of the previous Block; Hoegl et al., 2012)
  - Money was subtracted after incorrect reactions (reaction to a nogo trial or no reaction to a go-stimulus). Acoustic feedback was provided for positive and negative rewards.

*EEG implementation and processing*

- Brain electrical activity was recorded with sintered silver/silver chloride electrodes from 25 sites using an extended 10–20 system (10–20 system plus Fpz, CPz and mastoid electrodes; recording reference: FCz, ground electrode: CP2) using a standard EEG cap (EASYCAP, Herrsching, Germany).
- Electrooculogram electrodes were placed above and below the right eye and at the outer canthi.
- Filter bandwidth: 0.016–120 Hz (sampling frequency: 500 Hz)
- Impedances kept below 20 kΩ.
- Downsampling to 250 Hz (bandpass: 0.5 – 20 Hz; 24 db/Oct Butterworth filters and 50 Hz notch filter
- Ocular correction after Gratton, Coles & Donchin, 1983
- Referencing to linked mastoids
- Segmentation related to S2 stimulus:
  - cue segments: −1,850 to 100 ms
  - go/nogo segments: −150 to 1,150 ms
- The ERP components were determined in the averaged signal at the electrode with the highest amplitude: CNV (mean amplitude from −500 to 0 ms; Pz) and cue-P3 (maximum amplitude within −1,300 to −1,000 ms; Pz), go-P3 (maximum amplitude within 300–700 ms; Pz) and nogo-P3 (maximum amplitude within 300–700 ms; CPz). Performance data: Mean reaction time (RTM), the variability of reaction times (RTSTD), and the number of impulsivity errors (ERRimp) were considered.
